# Food Security Impacts of the COVID-19 Pandemic: Longitudinal Evidence from a Cohort of Adults in Vermont during the First Year

**DOI:** 10.3390/nu14071358

**Published:** 2022-03-24

**Authors:** Ashley C. McCarthy, Emily H. Belarmino, Farryl Bertmann, Meredith T. Niles

**Affiliations:** 1 Department of Nutrition and Food Sciences, University of Vermont, 109 Carrigan Drive, Burlington, VT 05405, USA; emily.belarmino@uvm.edu (E.H.B.); fbertman@uvm.edu (F.B.); mtniles@uvm.edu (M.T.N.); 2 Food Systems Program, University of Vermont, 109 Carrigan Drive, Burlington, VT 05405, USA; 3 Gund Institute for Environment, University of Vermont, 210 Colchester Ave., Burlington, VT 05405, USA

**Keywords:** COVID-19, food security, employment, food assistance, malnutrition

## Abstract

This study assessed changes in household food insecurity throughout the first year of the COVID-19 pandemic in a cohort of adults in the state of Vermont, USA, and examined the socio-demographic characteristics associated with increased odds of experiencing food insecurity during the pandemic. We conducted three online surveys between March 2020 and March 2021 to collect longitudinal data on food security, use of food assistance programs, and job disruptions during the COVID-19 pandemic. Food security was measured using the USDA six-item module. Among the 441 respondents, food insecurity rates increased significantly during the pandemic and remained above pre-pandemic levels a year after the start of the pandemic. Nearly a third (31.6%) of respondents experienced food insecurity at some point during the first year of the pandemic, with 53.1% of food-insecure households being classified as newly food-insecure. The odds of experiencing food insecurity during the pandemic varied based on socio-demographic factors. Households with children (OR 5.5, 95% CI 1.782–16.936, *p* < 0.01), women (OR 8.1, 95% CI 1.777–36.647, *p* < 0.05), BIPOC/Hispanic respondents (OR 11.8, 95% CI 1.615–85.805, *p* < 0.05), and households experiencing a job disruption (OR 5.0, 95% CI 1.583–16.005, *p* <0.01) had significantly higher odds of experiencing food insecurity during the first year of the COVID-19 pandemic, while respondents with a college degree (OR 0.08; 95% CI 0.025–0.246; *p* < 0.001) and household income of ≥USD 50,000 (OR 0.01; 95% CI 0.003–0.038; *p* < 0.001) had lower odds of experiencing food insecurity. These findings indicate that food insecurity continued to be a significant challenge one year after the start of the pandemic, which is important, given the adverse health impacts associated with food insecurity and health disparities among certain socio-demographic groups.

## 1. Introduction

A year after the start of the COVID-19 pandemic in the United States, the pandemic itself and the policies and restrictions put in place to reduce the spread of the virus continued to disrupt economies and labor markets, with serious implications for food insecurity. Food insecurity, defined as the lack of consistent physical and economic access to sufficient, safe, and nutritious food for an active and healthy lifestyle [1], is closely aligned with national and household economic conditions. Trends in food insecurity rates typically parallel those of unemployment, poverty, and food prices [2,3,4], though food insecurity can also result from non-economic drivers including limited physical access to food retailers and lack of transportation [5,6,7]. The COVID-19 pandemic likely exacerbated these challenges to food access due to safety concerns about shopping in stores, limited hours at food retailers, and changes in public transit access.

Food insecurity can lead to serious public health consequences. It is associated with numerous adverse physical and mental health outcomes, including heart disease, hypertension, diabetes, depression, an increased risk of mortality [8,9,10,11,12], and poorer diet quality [13,14]. Previous research has shown that healthcare use and costs are substantially higher among food-insecure adults [8,15,16,17]. Among households with children, food insecurity has also been linked with adverse educational and behavioral outcomes [10,18,19].

A number of peer-reviewed studies demonstrated increased food insecurity in the United States during the first months of the COVID-19 pandemic [20,21,22], but only a few studies have used a cohort model to examine changes in food security throughout the pandemic [20,23]. In the time since the data were collected in these early reports on food insecurity, new policies were implemented to provide economic relief to American households (e.g., stimulus checks, expansion of unemployment insurance, increased flexibility in federal food assistance programs, etc.), and many households experienced additional changes in income, unemployment, savings, and government assistance as the COVID-19 pandemic continued [24]. As a result, food insecurity prevalence has changed considerably throughout the course of the pandemic [25]. However, despite continual policy changes, cohort studies are less common in the current literature, but such studies are critical to assess how the same people’s food insecurity changed throughout the first year of the pandemic.

This study used longitudinal data to examine food insecurity during the first year of the COVID-19 pandemic amongst a cohort of respondents from Vermont, a U.S. state with a predominately rural population [26]. The cohort design allowed us to follow the same group of people throughout the first year of the pandemic to better understand fluctuations in household food security status, food assistance program participation, and job disruptions during this period, which would not be possible with a single point-in-time survey. Our objective was to answer the following research questions:

1. What was the trajectory of food insecurity during the first year of the COVID-19 pandemic among a cohort of respondents?
2. What socio-demographic factors and life experiences were associated with increased odds of experiencing food insecurity during the first year of the COVID-19 pandemic?
3. What factors, if any, contributed to the recovery from food insecurity during the first year of the COVID-19 pandemic?

## 2. Materials and Methods

### 2.1. Survey Development and Recruitment

We surveyed a cohort of adults in Vermont three times during the first year of the COVID-19 pandemic. The original survey [27] was developed with feedback from key state-level agencies and hunger relief organizations, as well as reviews of relevant literature [2,10], to measure food insecurity, food access challenges, and related concerns and experiences. When possible, this survey utilized existing validated questions and was updated after each round of data collection to add new relevant questions as the pandemic evolved. Using LimeSurvey [28], the instrument was piloted with 25 adults (18 years and older) from the target population of adults in Vermont.

**Figure A1:**
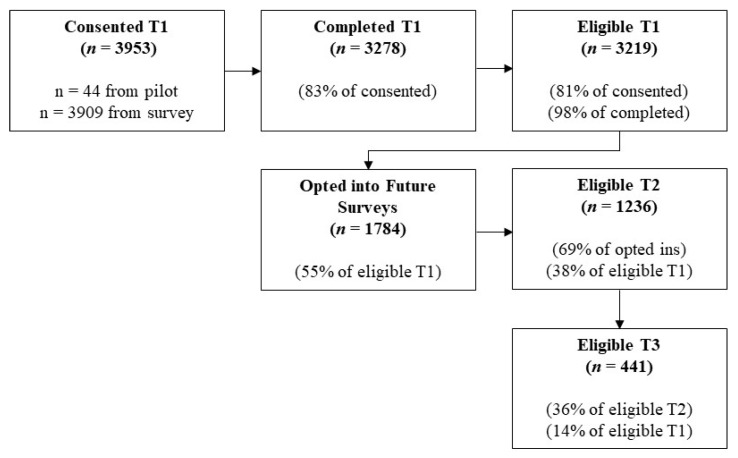
Breakdown of response to each survey.

The first survey ran online from 29 March to 12 April 2020, with a total of 3219 respondents. We used four methods for convenience sample recruitment: (1) paid advertisements via Front Porch Forum, a community-level listserv, which reaches approximately 2/3 of Vermont households [29]; (2) paid digital ads via Facebook to reach populations under-represented in Front Porch Forum (e.g., males, lower-income households); (3) listservs of community partners; (4) a University of Vermont press release and subsequent newspaper, radio, and television media. Respondents could opt-in to be contacted for future surveys at the first survey. We conducted two follow-up surveys with the same respondents in May/June 2020 and March/April 2021. The second survey ran online from 21 May to 4 June 2020, with a total of 1236 respondents. The third survey ran from 29 March to 21 April 2021. The first two surveys were conducted using LimeSurvey, and the third was conducted through Qualtrics [30]. Respondents with ZIP Codes outside Vermont and empty responses (i.e., people who consented but did not fill in any responses) were removed, leaving 441 eligible individuals who responded to all three surveys (Figure A1).

To measure household food security status, we adapted the U.S. Department of Agriculture’s (USDA) Household Food Security Survey Module: Six-Item Short Form [31] to ask about different time periods. The six questions are related to having enough food or enough money for food, being able to afford a balanced diet, and disrupted eating patterns (i.e., cutting the size of meals, skipping meals, going hungry). For example, one of the questions reads “Did you ever eat less than you felt you should because there wasn’t enough money for food?” with response options of “yes”, “no”, or “I don’t know”. We adapted the ends of the questions to ask about specific time periods. Across the three surveys, we assessed five timepoints. In the first survey, respondents were asked about food security both “in the year before the coronavirus outbreak” and “since the coronavirus outbreak”. The responses to the pre-pandemic questions about food security were retrospective and were answered at the same time as the questions about current food security. The start of the coronavirus outbreak was set as 8 March 2020, based on the first positive COVID-19 test result in Vermont. In the second survey, respondents were asked about food security “in the last 30 days” to reflect the time since the first survey. In the third survey, respondents were asked about food security both “since June 2020” (to reflect the time since the second survey) and “in the last 30 days”. We followed the procedures outlined in the USDA Six-Item Food Security Module [31] to code the responses to the six food security questions and calculate a food security status score for each respondent during each time period. We used a binary food security status score where two or more affirmative responses to the six questions is scored as food-insecure and fewer than two affirmative responses is scored as food-secure.

To determine respondents’ urban/rural classification, we used their ZIP Codes and the Rural–Urban Commuting Area (RUCA) codes [32] and the four-category classification scheme (urban, large rural, small rural, and isolated) created by the Rural Health Research Center [33]. We condensed this into a binary variable of urban and rural (which includes the categories large rural, small rural, and isolated from the four-category classification).

In addition to measuring food security status, the survey included questions related to food access challenges, use of food assistance programs, food purchasing behaviors, concerns about food access and availability, COVID-19 perceptions, and behaviors and demographics. Table A1 lists the specific questions used in this analysis, which primarily focused on understanding food security status, use of food assistance programs, and job disruptions. Future analyses will explore other questions in the survey.

### 2.2. Statistical Analysis

To examine differences in household food security during the first year of the pandemic, we created two core categories of respondents: (1) households with food security (*n* = 307, including households that were always food-secure during the first year of the COVID-19 pandemic as measured for all five time points) and (2) households with food insecurity (*n* = 134, including households that were food-insecure at any time during the first year of the COVID-19 pandemic). We further categorized food-insecure households into two groups: (1) households with consistent food insecurity (*n* = 61, including households that were food-insecure both in the year before the COVID-19 pandemic and anytime during the first year of the pandemic) and (2) households with new food insecurity (*n* = 69, including households that were food-secure before the pandemic, but food-insecure at some point during the first year of the pandemic). There were four food-insecure households that did not respond to the questions about food insecurity in the year prior to the COVID-19 pandemic, and therefore, we could not categorize them as consistently or newly food-insecure. We also categorized food-insecure households into two groups based on their food security status in March 2021, as measured with the 30-day instrument: (1) households that recovered by March 2021, meaning they were food-insecure at any point since the start of the pandemic but were food-secure in March 2021 (*n* = 48) and (2) households that were food-insecure at any point since the start of the COVID-19 pandemic and were still food-insecure in March 2021 (*n* = 78). There were eight food-insecure households that did not respond to the food insecurity questions in March 2021, and therefore, we could not categorize them as recovered or still food-insecure.

We used t-tests and chi-square tests to determine statistically significant differences between the groups described above. To determine the factors correlated with food insecurity during the first year of the COVID-19 pandemic, we used a random effects logistic regression model with panel data and calculated cluster robust standard errors to account for intra-subject correlation. We included a variable indicating which survey the observation was from to capture time factors that might have influenced food security given the pandemic-related changes that occurred over time during the study period. The coefficients are reported as odds ratios. To examine differences between groups (newly vs. consistently food-insecure; recovered vs. still food-insecure), we used multivariate logistic regression models, with coefficients reported in odds ratios. We used all available data and assumed any missing data were missing at random. A *p*-value ≤ 0.05 was considered statistically significant for all tests. All statistical analyses were conducted using Stata v17.1 [34].

## 3. Results

### 3.1. Demographic Characteristics of Respondents

The demographics of our respondents were comparable with Vermont state demographics on ethnicity and income distributions, but our respondents were more likely to have a college degree and to identify as female (Table 1; for full demographic results, see Table A2).

### 3.2. Food Insecurity, Job Disruptions, and Food Assistance Program Use

Food insecurity rates increased during the pandemic and remained above pre-COVID levels a year after the start of the pandemic (Table 2). It is important to note that the pre-COVID levels are based on retrospective responses to the questions about food insecurity. Among this cohort, 24.1% of respondents were classified as food-insecure in March 2020 (95% CI 20.0–28.2%) compared with 14.8% in the year before the COVID-19 pandemic (95% CI 11.5–18.2%), representing a 62.8% increase (*p* < 0.001) (Table A3). Compared with March 2020, food insecurity prevalence decreased in May/June 2020 to 17.4% (95% CI 13.9–21.0%) (*p* < 0.05) (Table A4) but then fluctuated throughout the first year of the pandemic. By March 2021, the food insecurity prevalence was 18.2% (95% CI 14.6–21.9%), representing a 24.5% decrease as compared with March 2020 (*p* < 0.05) but was 22.9% higher than pre-COVID levels (*p* = 0.18) (Table A5 and Table A6). Nearly a third (31.6%) of respondents experienced food insecurity at some point during the first year of the pandemic. Among those experiencing food insecurity during the first year of the pandemic, 46.9% also experienced food insecurity at some point in the year prior, while 53.1% were newly food-insecure. Of the respondents who experienced food insecurity at any point since the start of the pandemic, 61.9% were still classified as food-insecure in March 2021.

More than half of respondents (54.2%) reported suffering a job disruption (i.e., job loss, reduction in work hours or income, furlough) during the COVID-19 pandemic, and 18.7% were still reporting a job disruption in March 2021 (Table 3). The most common type of job disruption was a loss of hours or income (70.7%), followed by job loss (45.6%). The duration of these job disruptions varied, with 35.6% of respondents experiencing a job disruption lasting more than 6 months. More than one in five respondents (23.3%) received unemployment at some point since March 2020. Among those who reported job disruptions during the pandemic, 40.5% received unemployment.

We found that food-insecure respondents experienced job disruptions during the pandemic at higher rates than food-secure respondents, with 73.1% of food-insecure households (95% CI 65.5–80.7%) reporting a job disruption compared with 45.9% of food-secure households (95% CI 40.3–51.5%) (*p* < 0.001) (Table A7). More than three-fourths (78.7%) of consistently food-insecure households faced a job disruption. Food-insecure respondents continued to report higher rates of job disruption in March 2021 (95% CI 20.6–36.3%) compared with food-secure respondents (95% CI 10.4–18.3%) (*p* < 0.001) (Table A8). Additionally, food-insecure households received unemployment insurance (31.7%; 95% CI 23.4–40.0%) at higher rates than food-secure households (19.8%; 95% CI 15.2–24.3%) (*p* < 0.01) (Table A9).

**Figure 1:**
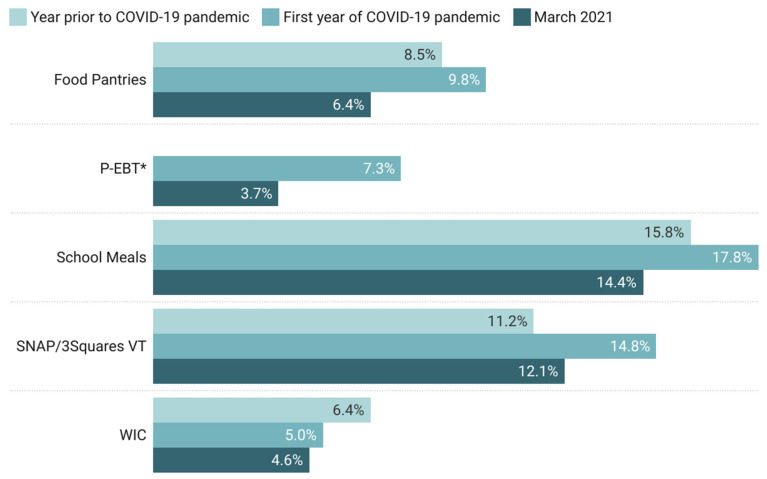
Change in food assistance program use during the first year of the COVID-19 pandemic. P-EBT did not exist prior to the pandemic. * Statistically significant difference (*p* ≤ 0.05).

Among our respondents, participation in food assistance programs increased during the first year of the COVID-19 pandemic compared with the year before, except for the Special Supplemental Nutrition Program for Women, Infants, and Children (WIC), though these differences were not statistically significant (Figure 1). However, by March 2021, participation in all programs had declined compared with time points measured earlier in the pandemic, with an 18.2% decrease in Supplemental Nutrition Assistance Program (SNAP/3SquaresVT), 49.3% decrease in Pandemic-EBT (P-EBT), 8.0% decrease in WIC, 19.1% decrease in school meal programs, and 34.7% decrease in the use of food pantries. Only the decline in P-EBT use was statistically significant, dropping from 7.3% at any time during the first year of the COVID-19 pandemic (95% CI 4.9–9.8%) to 3.7% in March 2021 (95% CI 1.9–5.4%) (*p* < 0.05) (Table A10). Food-insecure households (67.4%; 95% CI 59.3–75.5%) used food assistance programs at over three times the rate of food-secure households (16.3%; 95% CI 12.2–20.5%) (*p* < 0.001) (Table 3 and Table A11).

### 3.3. Factors Correlated with Food Insecurity

A random effects logistic regression model predicted the factors contributing to higher odds of experiencing food insecurity at any time during the first year of the COVID-19 pandemic (Table 4). Households that experienced any type of job disruption during the first year of the pandemic had greater odds of experiencing food insecurity (OR 5.03; 95% CI 1.583–16.005; *p* < 0.01). The odds of experiencing food insecurity were also higher among households with children (OR 5.49; 95% CI 1.782–16.936; *p* < 0.01), respondents who identified as BIPOC and/or Hispanic (OR 11.77; 95% CI 1.615–85.805; *p* < 0.05), and women (OR 8.07; 95% CI 1.777–36.647; *p* < 0.05). Older respondents (OR 0.05; 95% CI 0.012–0.180; *p* < 0.001) had lower odds of experiencing food insecurity compared with respondents under 63. Having a college degree (OR 0.08; 95% CI 0.025–0.246; *p* < 0.001) or a household income of ≥USD 50,000 (OR 0.01; 95% CI 0.003–0.038; *p* < 0.001) were also associated with reduced odds of household food insecurity.

We found no statistically significant differences (*p* < 0.05) using multivariate logistic regression models between newly and consistently food-insecure respondents (Table 5) and between respondents who were still food-insecure in March 2021 and those who had recovered (Table 6).

## 4. Discussion

This longitudinal study in Vermont documented a statistically significant increase in food insecurity compared with the year prior to the COVID-19 pandemic among a cohort of respondents. Furthermore, we show that while the food insecurity prevalence in March 2021 had decreased compared with the early months of the pandemic, rates remained higher than the year before the pandemic. This trend aligns with evidence from most other studies. For example, a longitudinal study conducted nationally found a reduced risk of food insecurity in November 2020 compared with March/April 2020 [23]. Adams et al. (2021) conducted surveys of a cohort of U.S. households with children in May and September 2020, finding that food insecurity increased in May 2020 compared with before the pandemic and then decreased in September 2020, but food insecurity remained above pre-pandemic levels [20]. However, a recent government report found no change in overall food insecurity prevalence in 2020 compared with 2019 [35], counter to most existing research.

We also demonstrated that certain demographic groups were at higher odds of experiencing food insecurity during the first year of the COVID-19 pandemic, including women, younger people (under 63), BIPOC/Hispanic respondents, people without a college degree, lower income households (<USD 50,000), households with children, and people who experienced a job disruption during the pandemic. These findings are consistent with other research on food insecurity during COVID-19 [22,35,36,37,38] and with research on food insecurity before the pandemic [2,3,4].

The increase in food insecurity documented here could have serious implications both in the short- and longer-term for physical and mental health [8,9,10,11]. Furthermore, health disparities among racial and ethnic minorities and people from lower socio-economic status are well-documented [39,40,41]. Given that our findings show that BIPOC/Hispanic respondents and lower income households were at greater risk for food insecurity during the first year of the COVID-19 pandemic, these groups may be particularly vulnerable to experiencing the associated health impacts in both the short and long term. Further research is needed to better understand how food insecurity during the COVID-19 pandemic has impacted diet quality and health, especially among socio-demographic groups that are at greater risk of food insecurity and adverse health outcomes.

Importantly, our results also show that a significant number of food-insecure households (32.6%) were not using federal food assistance programs or food pantries during the first year of the COVID-19 pandemic. Additionally, we found that only 40.5% of respondents who reported a job disruption during the pandemic received unemployment insurance. These findings demonstrate why we may have seen such a significant increase in food insecurity at the onset of the pandemic and the continued higher prevalence as compared with pre-pandemic periods. Particularly since more than half of our respondents were newly food-insecure, these households may have faced new barriers to receiving available assistance or may have gone without it altogether. Previous research has identified potential barriers to using food assistance programs, including stigma and administrative burden [42,43]. Lack of assistance may explain why food insecurity prevalence remained high, as many households in the United States live paycheck to paycheck and do not have the financial means to adapt to an economic shock [44]. Additional research is needed to continue understanding the barriers to using federal programs and to develop targeted solutions to ensure that households facing economic shocks can find necessary assistance.

Perhaps most surprising, our findings suggest that using federal programs designed to reduce the impact of the economic recession did not necessarily alleviate food insecurity during the first year of the pandemic when controlling for other demographic factors. Use of unemployment and/or use of food assistance programs did not predict recovery from food insecurity among our cohort. However, selection bias is a known issue when studying the impact of SNAP participation on food security [45,46], and it may have impacted our results. Additionally, two limitations of our work are the small sample size and the treatment of food insecurity as a binary outcome. Though the use of food assistance programs and/or unemployment did not necessarily move households out of food insecurity, it may have reduced the severity of the food insecurity they were experiencing and/or allowed households to reallocate money they would normally spend on food towards other essentials such as housing and healthcare. Future research using this longitudinal dataset will examine in more depth how various interventions, including federal and community food assistance programs and unemployment benefits, affected food insecurity outcomes in the first year of the COVID-19 pandemic, while treating food insecurity as a continuum.

Overall, our findings indicate that food insecurity continued to be a significant challenge one year after the start of the pandemic, despite loosened restrictions and new policies that aimed to provide economic relief. Trends from previous economic recessions show that it can take years after economic recovery begins for food insecurity rates to return to pre-recession levels [4,47] and that the financial hardships experienced during the pandemic will linger for some households even after they return to work, as they catch up on past due bills and replenish depleted savings. As some of the support programs come to an end (e.g., enhanced unemployment insurance, mortgage relief, eviction moratorium, student loan forbearance, etc.), it is imperative to continue monitoring the impact on food insecurity rates and continue providing assistance as the economy recovers, especially given the recent rise in food prices [48]. The recent update to SNAP benefits will increase the average SNAP benefit and could help alleviate some of the ongoing food insecurity challenges [49], though our findings show there is a gap in participation among food-insecure households.

This study provides important insights into the impact of COVID-19 on food insecurity throughout the first year of the pandemic. The study’s strengths include its longitudinal design, early administration, population-based assessment, and survey instrument addressing the multiple dimensions of food security. The limitations are partly rooted in the need to rapidly administer this survey in the early days of the pandemic in order to provide data that could be tracked over time. Though our respondent population matches statewide census statistics closely on many metrics, this was a convenience sample; further research is expanding these results using similar questions with representative samples across states and populations. It is worth noting that our observed overall rate of food insecurity prior to COVID-19 (14.8%) is above the most recently available state figure (9.6%) in 2019 [2]. There are multiple possible reasons for this. First, this is likely to be due, in part, to a higher than average number of female respondents and respondents in households with children; both groups have been documented, in Vermont and elsewhere, to have elevated rates of food insecurity [50]. Second, our measurement instrument for documenting food security, the USDA Six-Item Food Security Module, includes a subjective experience domain that measures concern about household food supplies. According to the local media [51], anxiety about household food supplies preceded the Stay Home/Stay Safe order and may explain the higher than expected level of food insecurity prior to COVID-19. Further, we used an internet-based survey, given the necessity of social distancing during COVID-19 and the need for a rapid response, which may limit the capacity of some people to participate, although 84.1% of adults in Vermont do have internet plans [52]. Another limitation is the wide confidence intervals for some variables (e.g., race/ethnicity, gender) in the logistic regression model predicting odds of food insecurity during the first year of the COVID-19 pandemic, which reduces the reliability of these estimates.

## 5. Conclusions

The prevalence of food insecurity increased during the first year of the COVID-19 pandemic and remained higher than pre-pandemic levels a year after the pandemic began. Our findings indicate that food insecurity continued to be a significant challenge for many people one year after the start of the pandemic, despite loosened restrictions and new policies that aimed to provide economic relief. Furthermore, the odds of experiencing food insecurity during the pandemic varied based on socio-demographic factors. Further research is needed to better understand how food insecurity during the COVID-19 pandemic has impacted diet quality and health, especially among socio-demographic groups that are at greater risk of food insecurity and adverse health outcomes.

This work also highlights a significant number of food-insecure households that were not using federal food assistance programs or food pantries during the first year of the COVID-19 pandemic, as well as fewer than half of respondents who reported a job disruption during the pandemic receiving unemployment insurance. Future research using this longitudinal dataset will further examine the characteristics of individuals and households that did and did not utilize these available services and will assess their relationship to food security and other outcomes.

## Figures and Tables

**Table 1:** Condensed characteristics of survey respondents, by food security category.

				Food Security Category			
Characteristic		Respondents (*n* = 441)	Vermont Population ^1^	Food-Secure (*n* = 307)	Food-Insecure (*n* = 134)	Consistently Food-Insecure (*n* = 61)	Newly Food-Insecure (*n* = 69)
		no. (%)	(%)	no. (%)			
Age	18 to 62	274 (62.1)	73.2	167 (54.4)	107 (79.9)	51 (83.6)	56 (81.2)
	63 and over	167 (37.9)	26.8	140 (45.6)	27 (20.1)	10 (16.4)	13 (18.8)
Gender	Female	347 (79.8)	50.7	231 (76.0)	116 (88.5)	52 (85.2)	63 (91.3)
	Not female	88 (20.2)	49.3	73 (24.0)	15 (11.5)	9 (14.8)	6 (8.7)
Race and ethnicity	White, non-Hispanic	407 (96.0)	92.6	289 (97.3)	118 (92.9)	56 (93.3)	62 (93.9)
	BIPOC and/or Hispanic	17 (4.0)	7.4	8 (2.7)	9 (7.1)	4 (6.7)	4 (6.1)
Education level	No college degree	95 (21.9)	52.2	41 (13.5)	54 (41.2)	31 (51.7)	22 (31.9)
	College degree	339 (78.1)	47.8	263 (86.5)	77 (58.8)	29 (48.3)	47 (68.1)
Household income (2020)	Less than USD 50,000	175 (42.1)	40.2	82 (28.2)	93 (74.4)	49 (81.7)	41 (67.2)
	USD 50,000 or more	241 (57.9)	59.8	209 (71.8)	32 (25.6)	11 (18.3)	20 (32.8)
Children in household	Yes	127 (29.7)	25.2	70 (23.3)	57 (44.9)	30 (51.7)	26 (38.2)
	No	301 (70.3)	74.8	231 (76.7)	70 (53.4)	28 (48.3)	42 (61.8)
Rural/urban classification	Urban	234 (54.2)	33.3	167 (55.5)	67 (51.1)	26 (42.6)	40 (58.0)
	Rural	198 (45.8)	66.7	134 (44.5)	64 (48.9)	35 (57.4)	29 (42.0)

Percentages may not total 100 due to rounding. Percentages are calculated using the number of respondents for that unique question and do not include missing data. ^1^ Data from the 2019 5-year American Community Survey.

**Table 2:** Food insecurity prevalence before and during the COVID-19 pandemic.

Time Period	Food-Insecure (%)
Year prior to COVID-19	14.8
Anytime during COVID-19	31.6
March 2020	24.1
May/June 2020	17.4
July 2020–February 2021	20.8
March 2021	18.2

**Table 3:** Respondent experiences with job disruptions, unemployment, and food assistance program use, by food security category.

			Food Security Category			
Variable		All Respondents	Food- Secure	Food- Insecure	Consistently Food-Insecure	Newly Food-Insecure
		%				
Any job disruption during COVID-19 pandemic		54.2	45.9	73.1	78.7	68.1
Experiencing a job disruption in March 2021		18.7	14.4	28.5	33.3	25.8
Type of job disruption ^1,2^						
	Job loss	45.6	39.7	54.1	62.5	44.7
	Loss of income/hours	70.7	73.8	66.3	64.6	70.2
	Furloughed	25.1	28.4	20.4	18.8	21.3
	Other	18.4	16.3	21.4	22.9	19.1
Length of job disruption ^1^						
	Less than 3 months	45.6	48.6	41.2	35.4	47.8
	3–6 months	19.8	19.3	20.6	22.9	15.2
	More than 6 months	35.6	32.1	38.1	41.7	37.0
Received unemployment		23.3	19.8	31.7	36.8	26.2
Used any food assistance program ^3^		31.7	16.3	67.4	73.8	59.7

^1^ Among respondents who reported a job disruption; ^2^ Respondents could indicate multiple types of job disruptions; ^3^ Includes SNAP, WIC, P-EBT, food pantries, and school meal programs.

**Table 4:** Random effects logistic regression predicting odds of food insecurity compared with food security during the first year of COVID-19.

Variable		Odds Ratio	Standard Error	*p* =	95% Confidence Interval	
Race/ethnicity						
	Non-Hispanic White	----------------------reference---------------------				
	BIPOC and/or Hispanic	11.771	11.930	0.015 *	1.615	85.805
Gender						
	Not female	----------------------reference---------------------				
	Female	8.070	6.230	0.007 **	1.777	36.647
Age						
	18–62	----------------------reference---------------------				
	63 and over	0.046	0.032	<0.001 ***	0.012	0.180
Households with children						
	No	----------------------reference---------------------				
	Yes	5.494	3.156	0.003 **	1.782	16.936
Income						
	Under USD 50,000	----------------------reference---------------------				
	USD 50,000 or more	0.010	0.007	<0.001 ***	0.003	0.038
Education						
	No college degree	----------------------reference---------------------				
	College degree	0.078	0.046	<0.001 ***	0.025	0.246
Rural/urban category						
	Rural	----------------------reference---------------------				
	Urban	0.668	0.365	0.461	0.229	1.948
Job disruption during COVID-19						
	No	----------------------reference---------------------				
	Yes	5.034	2.971	0.006 **	1.583	16.005
Survey occasion						
	Survey 1	----------------------reference---------------------				
	Survey 2	0.249	0.082	<0.001 ***	0.131	0.474
	Survey 3	0.522	0.171	0.048 *	0.274	0.993

* *p*-value < 0.05; ** *p*-value < 0.01; *** *p*-value < 0.001.

**Table 5:** Multivariate logistic regression predicting odds of being newly food-insecure during COVID-19.

Variable		Odds Ratio	Standard Error	*p* =	95% Confidence Interval	
Race/ethnicity						
	Non-Hispanic White	----------------------reference---------------------				
	BIPOC and/or Hispanic	1.168	0.976	0.853	0.227	6.009
Gender						
	Not female	----------------------reference---------------------				
	Female	2.232	1.507	0.234	0.594	8.382
Age						
	18–62	----------------------reference---------------------				
	63 and older	1.021	0.620	0.973	0.310	3.360
Households with children						
	No	----------------------reference---------------------				
	Yes	0.547	0.276	0.232	0.203	1.473
Income						
	Under USD 50,000	----------------------reference---------------------				
	USD 50,000 or more	1.564	0.829	0.399	0.553	4.421
Education						
	No college degree	----------------------reference---------------------				
	College degree	1.969	0.853	0.118	0.843	4.602
Rural/urban category						
	Rural	----------------------reference---------------------				
	Urban	1.875	0.796	0.139	0.816	4.310
Any job disruption during COVID-19						
	No	----------------------reference---------------------				
	Yes	0.547	0.276	0.232	0.204	1.471
Unemployment insurance						
	No	----------------------reference---------------------				
	Yes	0.950	0.440	0.912	0.384	2.353
Food assistance program use						
	No	----------------------reference---------------------				
	Yes	0.801	0.411	0.666	0.293	2.192

**Table 6:** Multivariate logistic regression predicting odds of recovering from food insecurity by March 2021.

Variable		Odds Ratio	Standard Error	*p* =	95% Confidence Interval	
Race/ethnicity						
	Non-Hispanic White	----------------------reference---------------------				
	BIPOC and/or Hispanic	0.897	0.746	0.896	0.176	4.581
Gender						
	Not female	----------------------reference---------------------				
	Female	1.055	0.703	0.935	0.286	3.895
Age						
	18–62	----------------------reference---------------------				
	63 and over	1.092	0.686	0.889	0.319	3.743
Households with children						
	No	----------------------reference---------------------				
	Yes	0.677	0.370	0.475	0.232	1.976
Income						
	Under USD 50,000	----------------------reference---------------------				
	USD 50,000 or more	2.288	1.232	0.124	0.797	6.573
Education						
	No college degree	----------------------reference---------------------				
	College degree	0.914	0.403	0.838	0.385	2.168
Rural/urban category						
	Rural	----------------------reference---------------------				
	Urban	1.471	0.645	0.378	0.623	3.474
Any job disruption during COVID-19						
	No	----------------------reference---------------------				
	Yes	1.551	0.848	0.422	0.531	4.526
Experiencing a job disruption in March 2021						
	No	----------------------reference---------------------				
	Yes	0.398	0.210	0.080	0.142	1.117
Unemployment insurance						
	No	----------------------reference---------------------				
	Yes	1.705	0.817	0.265	0.667	4.359
Food assistance program use						
	No	----------------------reference---------------------				
	Yes	0.833	0.455	0.738	0.286	2.427

## Data Availability

The survey instrument used in this study is publicly available and can be accessed at: https://dataverse.harvard.edu/dataverse/foodaccessandcoronavirus (accessed on 2 February 2022).

## Appendix A

**Table A1:** Complete list of variables, questions, and scales used in the analysis.

Variable	Question	Scale
Food-secure	Determined based on the responses to the U.S Household Food Security Survey Module: Six-Item Short Form. These households were not classified as food-insecure at any time during COVID-19.	Binary (1 = Food-secure, 0 = Food-insecure)
Food-insecure	Determined based on the responses to the U.S Household Food Security Survey Module: Six-Item Short Form. These households were food-insecure at any time during COVID-19, including newly food-insecure and consistently food-insecure households.	Binary (1 = Food-insecure, 0 = Food-secure)
Newly food-insecure	Determined based on the responses to the U.S Household Food Security Survey Module: Six-Item Short Form. These households were classified as not food-insecure during the year prior to COVID-19, but were classified as food-insecure at some point during the first year of the COVID-19 pandemic.	Binary (1 = Newly food-insecure, 0 = Consistently food-insecure)
Consistently food-insecure	Determined based on the responses to the U.S Household Food Security Survey Module: Six-Item Short Form. These households were classified as food-insecure both in the year prior to COVID-19 and at anytime during the first year of the COVID-19 pandemic.	Binary (1 = Consistently food-insecure, 0 = Newly food-insecure)
Recovered in March 2021	Determined based on the responses to the U.S Household Food Security Survey Module: Six-Item Short Form. These households were food-insecure at any point since the start of the pandemic, but were food-secure in March 2021.	Binary (1 = Recovered, 0 = Still food-insecure)
Still food-insecure in March 2021	Determined based on the responses to the U.S Household Food Security Survey Module: Six-Item Short Form. These households were food-insecure at any point since the start of the COVID-19 pandemic and were still food-insecure in March 2021	Binary (1 = Still food-insecure, 0 = Recovered)
Age	In what year were you born? (age determined by subtracting birth year from 2020)	Continuous
Age (binary)	Determined based on the responses to age question	Binary (1 = 63 and over, 0 = 18–62)
Household size	How many people in the following age groups currently live in your household (household defined as those currently living within your household, including family and non-family members)?	Number of people in each category (under 5, 5–17, 18–65, over 65)
Households with children	Whether respondent indicated any children in response to household size question	Binary (1 = Yes, 0 = No)
Gender	Which of the following best describes your gender identity?	1 = Male, 2 = Female, 3 = Transgender, 4 = Non-binary, 5 = Self describe
Gender (binary)	Determined based on the responses to gender question	Binary (1 = Female, 0 = Not female)
Race (binary)	Determined based on the responses to the questions "What is your race? Check all that apply"	Binary (1 = White, 0 = Non-white)
Ethnicity (binary)	Are you of Hispanic, Latino, or Spanish origin?	Binary (1 = Hispanic, 0 = Not Hispanic)
Race and ethnicity (binary)	Determined based on the responses to the race and ethnicity questions	Binary (1 = BIPOC and/or Hispanic, 0 = Non-Hispanic white)
Education	What is the highest level of formal education that you have?	Categorical (1 = Some high school, 2 = High school graduate/GED, 3 = Some college, 4 = Associates degree/technical school/apprenticeship, 5 = Bachelor’s degree, 6 = Postgraduate/professional degree
Education (binary)	Indication of associates degree/technical school/apprenticeship, bachelor’s degree, or postgraduate/professional degree in education question	Binary (1 = College degree, 0 = No college degree)
Household income	Which of the following best describes your household income range in 2020 before taxes?	1 = Less than USD 10,000, 2 = USD 10,000 to 14,999, 3 = USD 15,000 to 24,999, 4 = USD 25,000 to 34,999, 5 = USD 35,000 to 49,999, 6 = USD 50,000 to 74,999, 7 = USD 75,000 to 99,999, 8 = USD 100,000 to 149,999, 9 = USD 150,000 to 199,999, 10 = USD 200,000 or more
Household income (binary)	Determined based on the responses to household income question	Binary (1 = USD 50,000 or more, 0 = Less than USD 50,000)
Rural/urban classification	Determined based on the responses to the question "What is your ZIP code?" ZIP codes were categorized using the Rural-Urban Commuting Area codes and the four-category classification scheme.	Categorical (1 = Urban, 2 = Large rural, 3 = Small rural, 4 = Isolated)
Rural/urban classification (binary)	Determined based on rural/urban classification variable. Rural = large rural, small rural, and isolated.	Binary (1 = Urban, 0 = Rural)
SNAP participation	Determined based on the responses to question "Which of the following food assistance programs did your household use? Check all that apply." In each survey, the question was asked for the following reference periods: T1: in the year prior to COVID-19; since the coronavirus outbreak (March 11th) T2: in the last 30 days T3: since the coronavirus outbreak (March 2020); in the last 30 days	Binary (1 = Yes, 0 = No)
WIC participation		Binary (1 = Yes, 0 = No)
P-EBT participation		Binary (1 = Yes, 0 = No)
School meals participation		Binary (1 = Yes, 0 = No)
Food pantry participation		Binary (1 = Yes, 0 = No)
Any food assistance program participation (binary)	Determined based on the responses to the questions about individual program use	Binary (1 = Yes, 0 = No)
Job disruption during COVID-19 pandemic	Determined based on the responses to the questions: "Have you or anyone in your household experienced a loss of income, reduction in hours, furlough, or job loss since the COVID-19 outbreak? Check all that apply" and "In which month(s) did this job disruption occur?"	Binary (1 = Yes, 0 = No)
Job disruption in March 2021		Binary (1 = Yes, 0 = No)
Type of job disruption		Categorical (1 = Job loss, 2 = Loss of income/hours, 3 = Furlough, 4 = Other)
Length of job disruption		Continuous
Received unemployment	Have you received any money from these sources since the COVID-19 outbreak? Check all that apply.	Binary (1 = Yes, 0 = No)

**Table A2:** Full characteristics of survey respondents, by food security category.

				Food Security Category			
Characteristic		Respondents (*n* = 441)	Vermont Population ^1^	Food-Secure (*n* = 307)	Food-Insecure (*n* = 134)	Consistently Food-Insecure (*n* = 61)	Newly Food-Insecure (*n* = 69)
		no. (%)	(%)	no. (%)			
Age	18–34	49 (11.1)	27.6	29 (9.4)	20 (14.9)	11 (18.0)	9 (13.0)
	35–62	225 (51.0)	45.6	138 (45.0)	87 (64.9)	40 (65.6)	47 (68.2)
	63+	167 (37.9)	26.8	140 (45.6)	27 (20.1)	10 (16.4)	13 (18.8)
Household size	1–2	268 (62.8)	69.3	208 (69.1)	60 (47.6)	23 (39.7)	36 (53.7)
	2–4	126 (29.5)	25.0	82 (27.2)	44 (34.9)	18 (31.0)	26 (38.8)
	5 or more	33 (7.7)	5.6	11 (3.7)	22 (17.5)	17 (29.3)	5 (7.5)
Gender	Female	347 (79.8)	50.7	231 (76.0)	116 (88.5)	52 (85.2)	63 (91.3)
	Male	86 (19.8)	49.3	71 (23.4)	15 (11.5)	9 (14.8)	6 (8.7)
	Non-binary	2 (0.5)	--	2 (0.7)	0 (0.0)	0 (0.0)	0 (0.0)
	Transgender	0 (0.0)	--	0 (0.0)	0 (0.0)	0 (0.0)	0 (0.0)
	Other (self-describe)	0 (0.0)	--	0 (0.0)	0 (0.0)	0 (0.0)	0 (0.0)
Race	White	412 (97.4)	94.2	292 (98.3)	120 (95.2)	58 (96.7)	62 (95.4)
	Two or more races	4 (0.9)	2.0	0 (0.0)	4 (3.2)	1 (1.7)	2 (3.1)
	American Indian or Alaska Native	5 (1.2)	0.3	4 (1.3)	1 (0.8)	1 (1.7)	0 (0.0)
	Asian or Pacific Islander	0 (0.0)	1.7	0 (0.0)	0 (0.0)	0 (0.0)	0 (0.0)
	Black	2 (0.5)	1.4	1 (0.3)	1 (0.8)	0 (0.0)	1 (1.5)
Ethnicity	Not Hispanic or Latino	423 (98.4)	98.1	298 (98.7)	125 (97.7)	58 (96.7)	66 (98.5)
	Hispanic or Latino	7 (1.6)	1.9	4 (1.3)	3 (2.3)	2 (3.3)	1 (1.5)
Education level	Some high school (no diploma)	1 (0.2)	5.2	0 (0.0)	1 (0.8)	1 (1.7)	0 (0.0)
	High school graduate (incl. GED)	32 (7.4)	29.5	13 (4.3)	19 (14.6)	12 (20.0)	7 (10.1)
	Some college (no degree)	62 (14.3)	17.5	28 (9.2)	34 (26.2)	18 (30.0)	15 (21.7)
	Associate degree/technical school/apprenticeship	33 (7.6)	8.9	19 (6.3)	15 (11.5)	7 (11.7)	7 (10.1
	Bachelor’s degree	149 (34.3)	23.0	114 (37.5)	35 (26.9)	12 (20.0)	23 (33.3)
	Postgraduate/professional degree	157 (36.2)	15.9	130 (42.8)	27 (20.8)	10 (16.7)	17 (24.6)
Household income (2020)	Less than USD 10,000	13 (3.1)	4.8	1 (0.3)	12 (9.6)	8 (13.3)	4 (6.6)
	USD 10,000–14,999	25 (6.0)	5.0	9 (3.1)	16 (12.8)	8 (13.3)	7 (11.5)
	USD 15,000–24,999	39 (9.4)	9.1	13 (4.5)	26 (20.8)	14 (23.3)	11 (18.0)
	USD 25,000–34,999	40 (9.6)	9.1	21 (7.2)	19 (15.2)	9 (15.0)	10 (16.4)
	USD 35,000–49,999	58 (13.9)	12.2	38 (13.1)	20 (16.0)	10 (16.7)	9 (14.8)
	USD 50,000–74,999	71 (17.1)	18.7	53 (18.2)	18 (14.4)	6 (10.0)	11 (18.0)
	USD 75,000–99,999	66 (15.9)	14.0	59 (20.3)	7 (5.6)	3 (5.0)	4 (6.6)
	USD 100,000–149,999	66 (15.9)	16.0	62 (21.3)	4 (3.2)	1 (1.7)	3 (4.9)
	USD 150,000–199,999	27 (6.5)	5.6	24 (8.2)	3 (2.4)	1 (1.7)	2 (3.3)
	USD 200,000+	11 (2.6)	5.5	11 (3.8)	0 (0.0)	0 (0.0)	0 (0.0)
Children in household	Yes	127 (29.7)	25.2	70 (23.3)	57 (44.9)	30 (51.7)	26 (38.2)
	No	301 (70.3)	74.8	231 (76.7)	70 (53.4)	28 (48.3)	42 (61.8)
Rural/urban classification	Urban	234 (54.2)	33.3	167 (55.5)	67 (51.1)	26 (42.6)	40 (58.0)
	Large rural	60 (13.9)	20.1	37 (12.3)	23 (17.6)	10 (16.4)	13 (18.8)
	Small rural	51 (11.8)	18.0	34 (11.3)	17 (13.0)	10 (16.4)	7 (10.1)
	Isolated	87 (20.1)	28.6	63 (20.9)	24 (18.3)	15 (24.6)	9 (13.0)

Percentages may not total 100 due to rounding. Percentages are calculated using the number of respondents for that unique question and do not include missing data. ^1^ Data from the 2019 5-year American Community Survey.

**Table A3:** Results of a two-sided *t*-test for differences in food insecurity rates between the year prior to the COVID-19 pandemic and March 2020.

Variable	*n*	Mean	Std Error	Std Dev	95% Confidence Interval	
Food-insecure previous 12 months	432	0.148	0.017	0.356	0.115	0.182
Food-insecure March 2020	427	0.241	0.021	0.428	0.200	0.282
*p* = 0.0006						

**Table A4:** Results of a two-sided *t*-test for differences in food insecurity rates between March 2020 and May/June 2020.

Variable	*n*	Mean	Std Error	Std Dev	95% Confidence Interval	
Food-insecure March 2020	427	0.241	0.021	0.428	0.200	0.282
Food-insecure May/June 2020	436	0.174	0.018	0.380	0.139	0.210
*p =* 0.0153						

**Table A5:** Results of a two-sided *t*-test for differences in food insecurity rates between March 2020 and March 2021.

Variable	*n*	Mean	Std Error	Std Dev	95% Confidence Interval	
Food-insecure March 2020	427	0.241	0.021	0.428	0.200	0.282
Food-insecure March 2021	428	0.182	0.019	0.386	0.146	0.219
*p =* 0.0348						

**Table A6:** Results of a two-sided *t*-test for differences in food insecurity rates between the year prior to the COVID-19 pandemic and March 2021.

Variable	*n*	Mean	Std Error	Std Dev	95% Confidence Interval	
Food-insecure previous 12 months	432	0.148	0.017	0.356	0.115	0.182
Food-insecure March 2021	428	0.182	0.019	0.386	0.146	0.219
*p =* 0.1786						

**Table A7:** Results of a two-sided *t*-test for differences in reporting job disruptions during the COVID-19 pandemic between food-secure and food-insecure households.

Variable	*n*	Mean	Std Error	Std Dev	95% Confidence Interval	
Food-secure	307	0.459	0.028	0.499	0.403	0.515
Food-insecure	134	0.731	0.038	0.445	0.655	0.807
*p =* 0.0000						

**Table A8:** Results of a two-sided *t*-test for differences in reporting job disruptions in March 2021 between food-insecure and food-secure households.

Variable	*n*	Mean	Std Error	Std Dev	95% Confidence Interval	
Food-secure	306	0.144	0.020	0.351	0.104	0.183
Food-insecure	130	0.285	0.040	0.453	0.206	0.363
*p* = 0.0005						

**Table A9:** Results of a two-sided *t*-test for differences in use of unemployment insurance during the COVID-19 pandemic between food-secure and food-insecure households.

Variable	*n*	Mean	Std Error	Std Dev	95% Confidence Interval	
Food-secure	298	0.198	0.023	0.399	0.152	0.243
Food-insecure	123	0.317	0.042	0.467	0.234	0.400
*p =* 0.0085						

**Table A10:** Results of a two-sided *t*-test for differences in use of P-EBT during the COVID-19 pandemic.

Variable	*n*	Mean	Std Error	Std Dev	95% Confidence Interval	
First year of COVID-19 pandemic	438	0.073	0.012	0.261	0.049	0.098
March 2021	438	0.037	0.009	0.188	0.019	0.054
*p =* 0.0175						

**Table A11:** Results of a two-sided *t*-test for differences in use of food assistance programs during the COVID-19 pandemic between food-secure and food-insecure households.

Variable	*n*	Mean	Std Error	Std Dev	95% Confidence Interval	
Food-secure	306	0.163	0.021	0.370	0.122	0.205
Food-insecure	132	0.674	0.041	0.470	0.593	0.755
*p =* 0.0000						
